# Poly[hydroxybutyrate-co-hydroxyvalerate] and cellulose biocomposite membrane derived from sugarcane bagasse for Congo red dye and vegetable oil removal in water remediation

**DOI:** 10.1371/journal.pone.0336293

**Published:** 2025-11-06

**Authors:** Aophat Choonut, Alissara Reungsang

**Affiliations:** 1 Department of Biotechnology, Faculty of Technology, Khon Kaen University, Khon Kaen, Thailand; 2 Research Group for Development of Microbial Hydrogen Production Process from Biomass, Khon Kaen University, Khon Kaen, Thailand; 3 Academy of Science, Royal Society of Thailand, Bangkok, Thailand; University of South Africa, SOUTH AFRICA

## Abstract

Environmental contamination from textile dyes and oil residues poses a critical environmental and public health concern, highlighting the need for sustainable wastewater treatment strategies. A biocomposite membrane (BM) for the removal of Congo Red (CR) dye and vegetable oil (VO) from aqueous solutions was developed and evaluated separately for each pollutant in this study. Through a solvent casting technique, cellulose fibers (CF) from sugarcane bagasse (SCB) were successfully incorporated into a poly[hydroxybutyrate-co-hydroxyvalerate] (PHBV) matrix, as confirmed by FT-IR and SEM analyses. Exceptional adsorption efficiency was demonstrated by the BM, with 83.79% of CR dye (10 mg/L, pH 6.7) removed within 28,800 s and 95.15% of VO removed within 3,600 s, corresponding to a maximum oil adsorption capacity of 3.11 g-oil/g-sorbent, i.e., more than three times its own VO adsorption on the BM weight. The membrane exhibited good reusability, maintaining over 40% adsorption efficiency for CR dye after three cycles and over 50% efficiency for VO after five cycles. CR adsorption was found to follow the Langmuir model (R² = 0.9869) in isotherm studies, while VO adsorption aligned with the Freundlich model (R² = 0.9784). In kinetic analyses, CR adsorption was best described by the intra-particle diffusion model (R² = 0.9363), whereas VO adsorption followed the pseudo-second-order model (R² = 0.9999). The BM’s performance remained stable in the presence of ionic salts and synthetic wastewater. The significant potential of the BM as an environmentally friendly, cost-effective, and sustainable solution for long-term wastewater treatment applications through simultaneous dye and oil removal is highlighted by these findings.

## Introduction

Water pollution remains a pressing global concern, with the textile industry identified as one of the primary contributors to wastewater contamination. The widespread use of synthetic dyes in textile production, particularly carcinogenic compounds like Congo Red (CR), presents significant environmental and health risks [1,2]. Approximately 40% of dyes used in the industry are toxic and can degrade into harmful byproducts that pose threats to aquatic life [3]. In addition to dye pollution, oil contamination from both petrochemical spills [4,5] and household vegetable oil (VO) waste [6] further exacerbates environmental degradation. These pollutants persist in water, obstructing light penetration, reducing photosynthetic activity, and disrupting biodiversity, thereby threatening ecosystems and human health [3,7]. As a result, there is an urgent need for effective wastewater treatment methods and rapid responses to oil spills to mitigate these detrimental effects.

To address these challenges, various wastewater treatment techniques have been explored, including adsorption, biological, membrane filtration, ion exchange, biodegradation, catalytic ozonation and electrochemical oxidation for dye removal [3]. For oil removal, strategies such as in-situ burning [8], mechanical separation [9], adsorption [4] and bioremediation [10] have been employed. Among these, adsorption has garnered significant attention due to its efficiency, simplicity, low cost, and environmentally friendly nature [2,11–14]. Unlike other methods that may produce toxic residues or incur high operational costs [4,11,15], adsorption provides a practical solution for removing both dyes [13,14] and oils [4,5].

Recent advancements in adsorption materials have focused on developing sustainable adsorbents. Effective materials for dye removal include activated carbon [12,16], clay [17], biochar [18], sugarcane bagasse [19], banana leaves [20], walnut shell [21], zeolite [22], pinewood [23], chitosan [24], and biopolymers [3]. For oil adsorption, promising materials include cellulosic fibers [25], porous polymeric [26], polysiloxane sponges [27], carbon-based materials [28], hydrophobic fabrics [29], coated melamine sponges/foams [30] and biopolymers [31]. Despite the potential of these materials, several challenges remain, including high costs, limited durability, and reduced efficiency after multiple cycles. To address these issues, researchers have developed advanced materials for both dye and oil adsorption, such as acrylic-based membranes [32], Pt-doped titania pillared clay membranes [33], zeolite-based membranes [34], and cellulose-based [4,35–41] composites. Despite these innovations, the preparation of many adsorbents often involves complex processes and the use of hazardous chemicals, highlighting the need for more sustainable and environmentally friendly alternatives.

This study aims to address these challenges by developing a biocomposite membrane (BM) using cellulose derived from sugarcane bagasse (SCB) and poly(3-hydroxybutyrate-co-3-hydroxyvalerate) (PHBV) through a solvent casting method. This approach offers a simple, scalable fabrication process that operates under mild conditions, avoiding the use of hazardous chemicals, making it both cost-effective and sustainable. Cellulose from SCB is a cost-effective, biodegradable [42], and hydrophilic material [43], making it a promising choice for dye wastewater treatment and supporting circular economy principles. Furthermore, recent studies have highlighted the effectiveness of cellulose-based materials in wastewater treatment due to their high surface area and adsorption capacity. PHBV is a biodegradable, non-hazardous, and cost-effective biopolymer [44], with hydrophobic properties [45] that enhance oil adsorption and improve the membrane’s mechanical strength [46], thereby representing a sustainable alternative to conventional synthetic polymers. This combination enables the BM to effectively capture both polar and nonpolar contaminants, a feature that has not been widely explored in wastewater treatment applications.

The main aim of this investigation was to develop and characterize a novel BM fabricated from SCB cellulose fibers and PHBV for the removal of CR dye and VO from contaminated water, with adsorption experiments conducted separately for each pollutant. The membrane’s adsorption performance was evaluated through adsorption isotherms, kinetics, point of zero charge, and stability in the presence of ionic salts and synthetic wastewater. The results demonstrated high adsorption capacity, satisfactory reusability, and notable stability under various conditions. These attributes, together with the membrane’s biodegradability and cost-effectiveness, highlight its potential as a sustainable alternative to conventional adsorbents for treating industrial effluents containing organic dyes or oils.

## Materials and methods

### Chemicals and feedstock

PHBV biopolymer (C₉H₁₈O₆), high purity confirmed by NMR spectrum, polyhydroxybutyrate (PHB) content: 92 mol%, polyhydroxyvalerate (PHV) content: 8 mol%) was purchased from Merck KGaA (Darmstadt, Germany). SCB was supplied by Mitr Phu Kieo Sugar Mill (Mitr Phol Group, Chaiyaphum, Thailand). The SCB was washed to remove impurities and dried at 80°C for 86,400 s. It was then cut into 1 cm pieces and stored in an airtight container.

All chemicals and solvents used were of analytical reagent grade, sourced from RCI Labscan (MP Impex Co., Ltd., Thailand), including sodium hydroxide (NaOH, ≥ 99.0%), sodium chlorite (NaCl, ≥ 99.0%), sodium acetate (CH₃COONa, ≥ 99.5%), acetic acid (CH₃COOH, ≥ 99.8%), hydrochloric acid (HCl, ≥ 99.8%), and chloroform (CHCl₃, ≥ 99.8%), calcium chloride (CaCl₂ ≥ 99.8%), sodium sulfate (Na₂SO₄ ≥ 99.0%), magnesium sulfate (MgSO₄ ≥ 98.0), sodium hydrogen phosphate (Na₂HPO₄ ≥ 98.0), and sodium carbonate (Na₂CO₃ ≥ 98.0). CR dye was purchased from KemAus™ (Australia), and VO was obtained from a local department store in Khon Kaen, Thailand.

### Preparation of biocomposite membrane

For cellulose fiber (CF) preparation, the SCB was immersed in a 4% NaOH solution at 90°C for 7,200 s with continuous stirring. After washing with tap water until the pH reached neutral (7.0), the material was treated with a 2% NaCl solution and acetate buffer at 90°C for 14,400 s [47,48]. Following another washing step, the material was air-dried and ground into 3–5 mm fibers.

To confirm that the CF prepared from SCB was indeed cellulose, its dyeing capacity was evaluated using CR dye, which specifically binds to cellulose [49,50]. The CF was mixed with CR dye at a concentration of 5 mg/L (pH 6.7) and incubated at 30°C with an agitation speed of 120 rpm for 21,600 s. The success of CF preparation from SCB was confirmed by evaluating its enhanced adsorption capacity compared to SCB.

For BM preparation, 2.5 g of PHBV was dissolved in 50 mL CHCl_3_ at 50°C for 43,200 s in a sealed Duran bottle to prevent solvent evaporation. Subsequently, 1.5 g of CF was gradually added to the PHBV/CHCl_3_ solution and stirred at 50°C for 86,400 s, as modified by Sangkharak et al. [47]. The resulting mixture was then poured into a glass container to form the membrane. The membrane was stored at room temperature (30 ± 2°C) for 86,400 s before characterization using Fourier-transform infrared spectroscopy (FTIR) and scanning electron microscopy (SEM).

### Pollutant removal performance

#### Congo red dye removal.

The CR dye concentrations (10–100 mg/L) were selected to represent typical aqueous dye pollution levels [51,52]. The BM sheet measuring approximately 3.65 × 3.65 cm (≈0.5 g) [51,53] was immersed in 20 mL of water containing CR dye at 10 mg/L. The pH of the solution was adjusted to 6.7 using 0.1 mol/L HCl or NaOH solutions, as dye precipitation may occur at pH values outside this range. The mixture was agitated at 150 rpm for 43,200 s at room temperature (30 ± 2°C). Samples were collected at intervals of 1,800–43,200 s to assess dye removal efficiency. The removal was monitored by measuring absorbance at 488 nm using a UV-Vis spectrophotometer and calculated using Equation 1 [50,51].


$$\text{D }=\left[\frac{\text{A}-\text{B}}{\text{A}}\right]\times \;\text{1}\text{0}\text{0}$$ (1)

Where: **D** is the dye removal (%), **A** is the initial absorbance at time 0, and **B** is the absorbance at time t.

To evaluate the BM’s maximum dye adsorption capacity, CR dye concentrations ranging from 10 to 100 mg/L were used with a contact time of 28,800 s. For reusability assessment, the BM underwent repeated adsorption cycles. After each initial adsorption cycle (10 mg/L dye, 28,800 s), the BM was regenerated by immersion in a 5 mol/L HCl solution for 7,200 s [47], followed by five rinses with distilled water and drying at room temperature. The BM was weighed before and after each regeneration cycle using an analytical balance (Precisa XB220A, Max 220 g, e = 0.001 g, Min = 0.01 g, d = 0.0001 g, Precisa Gravimetrics AG, Switzerland to confirm that no significant mass loss occurred. These regeneration-reuse cycles continued until the dye removal efficiency decreased below 50%.

#### Vegetable oil removal.

To determine the oil absorption capacity, the BM was initially weighed before being immersed in VO. A higher VO concentration (4,000 g/L) was used due to its hydrophobicity and density, ensuring measurable adsorption [54]. To determine the oil absorption capacity, the BM was initially weighed before being immersed in VO. A BM sheet measuring 2 × 2 cm (≈0.15 g) [55] was weighed and immersed in 25 mL of water containing VO at 4,000 g/L. The mixture was agitated at 150 rpm for 28,800 s at room temperature (30 ± 2°C). At the following time intervals: 1,800–28,800 s, BM samples were taken to measure the weight changes due to oil adsorption. Before weighing, the BM was dried at 50°C for 86,400 s to remove any moisture. The weight was determined using an analytical balance (Precisa XB220A, Max 220 g, e = 0.001 g, Min = 0.01 g, d = 0.0001 g). Replicate measurements were performed to confirm data reliability. The oil adsorption efficiency was calculated using Equation 2, modified from Sudesh et al. [31] and Al-Najar et al. [56].


$$\text{O}=[\text{A}-\left(\frac{\text{C}-\text{B}}{\text{A}}\right)\times \text{ 100}$$ (2)

Where: **O** is the oil removal (%), **A** is the initial weight of the oil used in the test (g), **B** is the weight of the BM before adsorption (g), and **C** is the weight of the BM after adsorption (g)

To assess the reusability of the BM, the BM underwent a regeneration process after 3,600 s of oil absorption. The BM was immersed in a 5% detergent solution (common laundry powder) for 86,400 s, with continuous shaking at 150 rpm at room temperature (30 ± 2°C). Afterwards, the BM was rinsed thoroughly with tap water until bubble-free and air-dried at room temperature (30 ± 2°C) for 86,400 s [31]. The BM was weighed before and after each regeneration cycle to ensure that no significant material loss occurred. The regeneration and reuse cycle was repeated until the oil adsorption efficiency decreased to below 50%.

To evaluate the oil retention capacity, the BM was compared with two commercial oil adsorbents: (1) sample No. 1, Scott multipurpose paper, and (2) sample No. 2, Maxmo multipurpose paper. The BM and commercial oil adsorbents (2 × 2 cm each) were weighed before VO was added dropwise until excess oil leakage was observed (modified from Sudesh et al. [31]). After 3,600 s at room temperature, a 2 kg water bottle wrapped in foil was gently placed on each sample for 60 s to simulate mild pressure conditions and to ensure uniform contact between the oil and the adsorbent surface, allowing for accurate measurement of the oil retention capacity. The BM and commercial oil adsorbent samples were then weighed again. The oil retention capacity was calculated using Equation 3.


$$\text{Q }=\left[\frac{{\text{C}}_{\text{t}}-{\text{C}}_{\text{0}}}{{\text{C}}_{\text{0}}}\right]\times \text{ 100}$$ (3)

**Q** is the oil retention capacity (g of oil adsorbed per g of adsorbent), **C**_**0**_ is the initial weight of the adsorbent (g), and **C**_**t**_ is the weight of the adsorbent after oil absorption (g)

### Kinetic and isotherm study

The adsorption behavior of the BM was evaluated using both the Freundlich and Langmuir isotherm models. Additionally, the kinetics of adsorption on BM were investigated using the pseudo-first-order (PFO), pseudo-second-order (PSO), and intra-particle diffusion (IPD) models. Both isotherm and kinetic calculations were based on the equations and calculations from Radoor et al [57].

#### Isotherm.

**Langmuir model.** The Langmuir model is based on the assumption that the surface of the adsorbent is homogeneous, with a finite number of energetically equivalent adsorption sites. This model suggests that adsorption occurs uniformly across the surface of the adsorbent, with no interaction between the adsorbed molecules. The model implies that each adsorption site can hold only one molecule, and once a site is occupied, no further adsorption can occur at that location. The Langmuir adsorption isotherm is typically represented by the following equation:


$${\text{q}}_{\text{e}}=\frac{{\text{q}}_{\text{m}}\;{\text{K}}_{\text{L }}{\text{C}}_{\text{e}}}{\text{1}\;+\;{\text{K}}_{\text{L }}{\text{C}}_{\text{e}}}$$ (4)

Where: **q**_**e**_ corresponds to the equilibrium amount of adsorbate or adsorption capacity (mg/g), and **C**_**e**_ represents the equilibrium concentration of adsorbate (mg/L). **K**_**L**_ and **q**_**m**_ represent the Langmuir constant and are related to adsorption energy and adsorption capacity, respectively. The linear form of the Langmuir isotherm model is expressed as follows:


$$\frac{{\text{C}}_{\text{e}}}{{\text{q}}_{\text{e}}}\;=\;\left(\frac{{\text{C}}_{\text{e}}}{{\text{q}}_{\text{m}}}\right)\;+\;\left(\frac{\text{1}}{{\text{K}}_{\text{L}}*\;{\text{q}}_{\text{m}}}\right)$$ (5)

The plot of **C**_**e**_**/q**_**e**_ vs **C**_**e**_ results in a straight line with **q**_**m**_ as slope and **K**_**L**_ as intercept.

**Freundlich model.** The Freundlich isotherm is an empirical model that assumes a non-uniform distribution of adsorbate on the surface of the adsorbent. It is commonly used to describe multilayer adsorption, where adsorption occurs at various sites with different affinities. The non-linear form of the Freundlich isotherm is given by:


$${\text{q}}_{\text{e}}\;=\;{\text{K}}_{\text{F}}\times \;{\text{C}}_{\text{e}}^{\text{1}/\text{n}}$$ (6)

In the Freundlich model, **q**_**e**_ represents the amount of CR dye and VO adsorbed onto the BM at equilibrium (mg/g), and **C**_**e**_ is the equilibrium concentration of CR dye and VO in the solution (mg/L). The constants **K**_**F**_ and **1/n** correspond to the adsorption capacity and adsorption intensity (indicating surface homogeneity), respectively. These values are determined from the slope and intercept of the plot of **ln(q**_**e**_) versus **ln(C**_**e**_). The value of **1/n** indicates the favorability of the adsorption process. If **1/n** is between 0 and 1, the adsorption is considered favorable. A value of **1/n** greater than 1 or equal to 0 suggests that the adsorption is either irreversible or unfavorable, respectively. The linear form of the Freundlich isotherm is represented as follows:


$$\text{In}({\text{q}}_{\text{e}})\;=\text{ In }{\text{K}}_{\text{F}}+\;\frac{\text{1}}{\text{n}}\;\times \text{ In}({\text{C}}_{\text{e}})$$ (7)

#### Adsorption kinetics.

Adsorption kinetics gives an idea about the controlling mechanism of the adsorption process (chemical reaction, diffusion, and mass transfer process) and provides information regarding adsorption rate and adsorbate residue time. To investigate the kinetics of CR dye and VO adsorption on the BM, PFO, PSO, and IPD models were employed. The linear form of PFO and PSO is given below.

**PFO models:**


$$\text{log}\left({\text{q}}_{\text{e }}-\;{\text{q}}_{\text{e}}\right)\;=\text{ log}{\text{q}}_{\text{e}}-\;\frac{{\text{K}}_{\text{1}}\text{t}}{\text{2}.\text{303}}$$ (8)

**PSO models:**


$$\frac{\text{t}}{{\text{q}}_{\text{e}}}\;=\frac{\text{1}}{{\text{K}}_{\text{2}}{\text{q}}_{\text{e}}^{\text{2}}}\;+\;\frac{\text{t}}{{\text{q}}_{\text{e}}}$$ (9)

where: **K**_**1**_ (s^−1^) is the PFO rate constant, **K**_**2**_ (g/mg s^−1^) is the PSO rate constant, **q**_**t**_ (mg/g) is the adsorption capacity at time **t** (s) and **q**_**e**_ (mg/g) is the adsorption capacity at equilibrium, respectively, which is determined from the slope of **log (q**_**e**_** − q**_**t**_) versus time and **t/q**_**t**_ versus time of a linear plot. According to the intra-particle diffusion model, the kinetics of adsorption involves diffusion of the adsorbent into the pores of the adsorbent and is represented as:


$${\text{q}}_{\text{t}}\;=\;{\text{K}}_{\text{id}}{\text{t}}^{\text{1}/\text{2}}$$ (10)

Where: **K**_**id**_ and **C** are intraparticle constants and are estimated from the slope and intercept of the plot between **q**_**t**_ vs **t**^**1/2**^.

### Characterization of biocomposite membrane

#### Characterization before and after adsorption of biocomposite membrane by FTIR and SEM.

The BM was characterized before and after dye and oil adsorption using SEM (Thermo Fisher Scientific, Model: Quattro-S E-SEM) and FTIR (Tensor II, Bruker, Germany, coupled with a Platinum ATR accessory). The BM was cut into 0.5 × 0.5 cm pieces for consistency. SEM was used to observe changes in surface morphology, such as roughness and porosity, which affect adsorption capacity. FTIR analysis in the range of 4,000–400 cm ⁻ ¹ was performed to identify changes in functional groups on the BM surface, indicating interactions with dye or oil during adsorption. FTIR spectra were obtained from representative samples before and after adsorption. Peak intensity changes were calculated to provide qualitative insights into functional group involvement during the adsorption process. Percentage changes represent relative differences in peak intensities between pristine and adsorption-treated samples.

#### Characterization of pH at the point of zero charge (pH_PZC_) of biocomposite membrane.

The pH_PZC_ of the BM was determined to understand its surface charge behaviour, which influences adsorption properties. The pH_PZC_ was measured using the 11-point methodology [58], in which 11 suspensions of BM (1.0 g/L) were prepared in aqueous NaCl solution with initial pH adjusted from 2 to 12 using 0.1 mol/L HCl or NaOH. The suspensions were stirred at 80 rpm for 24 h at 25°C [59]. after which the final pH was recorded using a pH meter (Model SP-2300). The pH_PZC_ was determined as the pH at which the difference between initial and final pH (ΔpH) was zero.

### Influence of ionic salts and synthetic wastewater on adsorption

#### Ionic salts on adsorption.

The effects of different ionic salts (NaCl, CaCl_2_, Na_2_SO_4_, MgSO_4_, and Na_2_HPO_4_) on the adsorption of CR dye and VO were investigated [12,60]. The experiments were conducted in two solutions: 20 mL of CR dye solution (10 mg/L, pH 6.7) and 25 mL of VO-contaminated water (4,000 mg/L). To simulate the ionic strength and composition found in wastewater, each salt was separately added to the solutions at a concentration of 0.2% (w/v) [61]. The mixtures were agitated at 150 rpm for 28,800 s for dye adsorption and 3,600 s for oil adsorption at room temperature (30 ± 2°C). The effects of the ionic salts on the adsorption of CR dye and VO onto the BM were calculated using Equations 1 and 2, respectively.

#### Synthetic wastewater on adsorption.

Synthetic-dyed wastewater was prepared by modifying the method from Kheddo et al. [62], where CR dye was dissolved in water at a concentration of 10 mg/L. To replicate typical wastewater conditions, Na_2_CO_3_ (20 g/L) and Na_2_SO_4_ (30 g/L) were added, and the components were mixed and agitated to ensure thorough dissolution, simulating the ionic strength and composition of wastewater. The resulting solution, containing both the dye and salts, was used to create synthetic dyed wastewater for adsorption studies. For the oil adsorption tests, a similar composition (without dye) was used, with oil added at a concentration of 4,000 mg/L. The effects of the synthetic wastewater on the adsorption of CR dye and VO onto the BM were investigated by collecting samples at different time intervals, under agitation at 150 rpm and room temperature (30 ± 2°C). The adsorption data were then calculated using Equations 1 and 2, respectively.

### Statistical analysis

All experiments were conducted in triplicate, with results expressed as mean ± standard deviation (SD). Statistical analysis was performed using one-way analysis of variance (ANOVA) at a 95% confidence interval through Microsoft Excel 2010’s Add-in statistical package.

## Results and discussion

To confirm the successful extraction of CF from SCB (in Section 2.2), a dye adsorption test was conducted. The results showed a significant increase in CR dye uptake by CF, with an adsorption rate of 92.0 ± 1.3%, compared to only 45.4 ± 2.1% for SCB. This indicates that lignin removal and enhanced cellulose availability effectively improved adsorption efficiency. Thus, the substantial improvement in dye adsorption capacity confirms that the CF preparation process successfully modified the SCB structure, making it more suitable for pollutant removal. With the successful extraction of CF confirmed, the next step involves its incorporation with PHBV to fabricate a BM, as detailed in the following section.

### Stepwise preparation of biocomposite membrane

**Fig 1:**
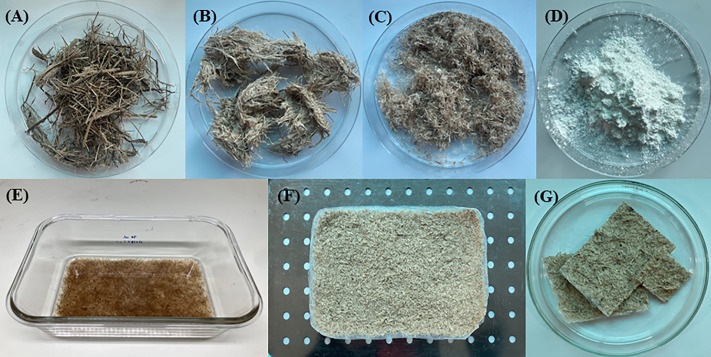
The preparation of biocomposite membrane (BM) through a simple process. (A) sugarcane bagasse (SCB), (B) SCB after pretreatment, (C) cellulose fibers (CF) extracted from SCB, (D) poly[hydroxybutyrate-co-hydroxyvalerate] (PHBV), (E) a mixed solution of BM from CF and PHBV by solution casting, (F) BM obtained from the CF and PHBV, and (G) the final BM product used in this study.

The preparation process of BM, shown in **Fig 1**, demonstrates the stepwise transformation from raw SCB to the final BM. The initial SCB material (**Fig 1A**) appears as coarse, fibrous strands, which undergo processing to extract CF (Fig 1A–1C). These extracted CF demonstrate superior strength and stretchability compared to commercial rayon fibers [63]. The progression from Fig 1B to 1C shows the gradual refinement of the fibers, with the material becoming more uniformly textured and finer in structure. PHBV, shown in **Fig 1D** as a white powdery substance, provides the polymer matrix for the composite, offering advantages due to its biodegradable nature and flexible, porous structure that enhances surface area and adsorption capabilities [64]. The solution casting process (**Fig 1E**) shows the homogeneous mixing of CF and PHBV in chloroform, resulting in a uniform brown solution that indicates successful dissolution and blending of both components. The combination of cellulose [43] and PHBV [44,45] creates a synergistic system that enhances the BM’s adsorption capacity by targeting both polar and non-polar molecules. The final BM product (Fig 1F–1G) appears as a uniform, light-colored sheet with consistent thickness and texture, suggesting effective integration of the CF within the PHBV matrix. This integration creates a microporous structure that facilitates ion exchange and adsorption [65], effectively removing various contaminants [66,67]. The sheet’s appearance indicates good dispersion of the CF throughout the polymer matrix, which is crucial for optimal performance in pollutant removal applications. Additionally, the interaction between cellulose and PHBV enhances the material’s mechanical and thermal properties [46,68], contributing to the resilience of the BM in environmental applications such as waste treatment or pollutant adsorption.

#### Morphological analysis of biocomposite membrane for pollutant adsorption.

**Fig 2:**
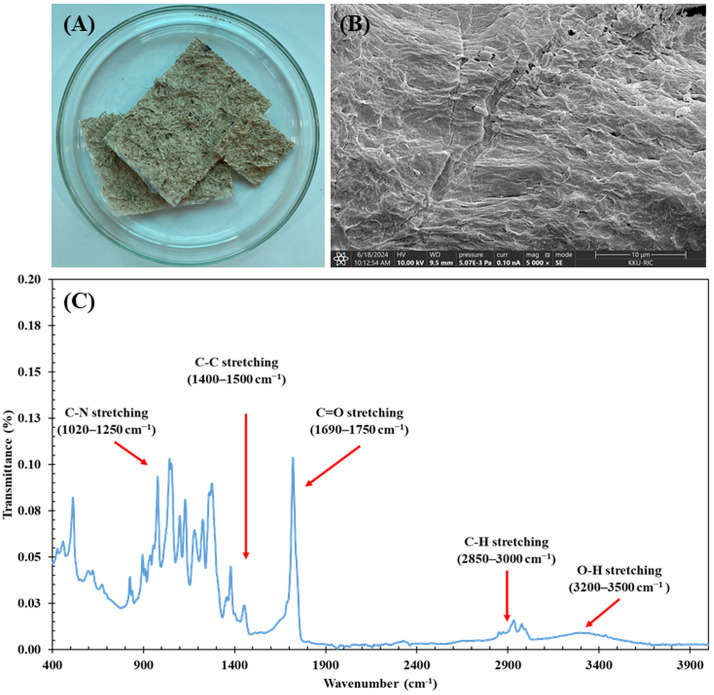
Physical characteristics of the biocomposite membrane (BM). (A) BM sheet used in the experiment, (B) morphology of the BM analyzed with SEM (5,000x), and (C) functional groups of BM by FTIR.

The BM demonstrates strong adhesion between cellulose and PHBV components (**Fig 2A**), which enhances their strength and durability. SEM analysis reveals a rough surface texture created by CF cross-linking, characterized by a generally smooth surface with minor cracks and visible pores or dimples (**Fig 2B**). These structural features make the BM particularly effective as a biosorbent material for dye and oil removal in wastewater treatment. This morphology aligns with previous research demonstrating that cellulose-based materials, when combined with biopolymers like PHBV, exhibit enhanced surface properties, including roughness and porosity that facilitate pollutant adsorption [69,70]. Similar findings have been reported in studies by Radoor et al. [57], Rawat et al. [71], and Abou Alsoaud et al. [72], where similar structures were found to enhance the adsorption capacities of various composites in wastewater treatment. Further confirmation of the biocomposite’s chemical characteristics was obtained through FTIR analysis. The FTIR spectra (**Fig 2C**) show key functional groups, such as hydroxyl (-OH) and carboxyl (-C-H) groups, which are essential for interacting with pollutants [73,74], contributing to the biocomposite’s ability to adsorb contaminants. This is consistent with findings from Al-Gethami et al. [75] and Lindman et al. [76], and other studies on biocomposites [41,77], where similar functional groups were found to play a significant role in pollutant removal. These findings further support the critical role of both surface morphology and chemical composition in the adsorption process. The combination of rough surface features, functional group availability, and hydrophilic properties enhances the overall performance of the BM in wastewater treatment applications.

#### The pH_PZC_ of biocomposite membrane for pollutant adsorption.

**Fig 3:**
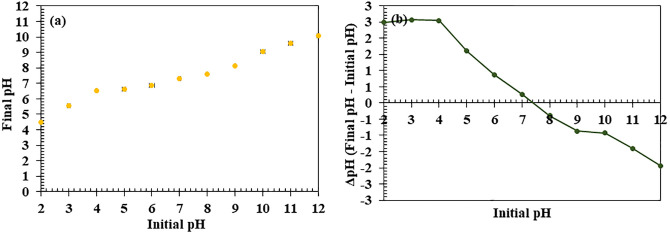
Surface chemistry analysis of the biocomposite membrane (BM). (A) initial pH vs. final pH and (B) initial pH vs. ΔpH (change in pH).

In this study, the pH_PZC_ of the BM was determined to be 7.29 ± 0.04, indicating a neutral surface charge (**Fig 3A**). From the net change in pH (ΔpH) versus initial pH plot (**Fig 3B**), it was observed that the ΔpH decreased as the initial pH increased. The pH_PZC_ is the point where ΔpH = 0, which corresponds to an initial pH of approximately 7.29. Below the pH_PZC_, the material exhibits a positive charge due to protonation of functional groups [78] like hydroxyl (-OH) and carboxyl (-COOH) [76], while at higher pH, deprotonation leads to a negative charge [79]. This behavior allows the membrane to interact with both cationic and anionic species, enhancing its versatility in adsorbing various pollutants, including CR dye. During adsorption experiments (pH 6.7), the membrane’s positive charge improves the adsorption of negatively charged pollutants [59,80,81]. Specifically, CR is an anionic azo dye carrying negatively charged sulfonate (-SO₃⁻) groups at neutral pH. The electrostatic attraction between the positively charged BM surface and the anionic CR molecules significantly contributes to the adsorption process, along with additional interactions such as hydrogen bonding and π–π stacking. Although VO is non-polar, its adsorption is facilitated by hydrophobic interactions with PHBV’s hydrophobic regions [45], enhanced by the positively charged surface at neutral pH. The membrane’s ability to adapt its surface charge based on pH makes it ideal for dynamic environmental conditions, optimizing performance for a wide range of pollutants. These insights offer a foundation for predicting adsorption behavior and optimizing conditions for large-scale applications, such as wastewater treatment and pollution control.

### Biocomposite membrane performance in wastewater treatment

#### Congo red dye removal efficiency.

**Fig 4:**
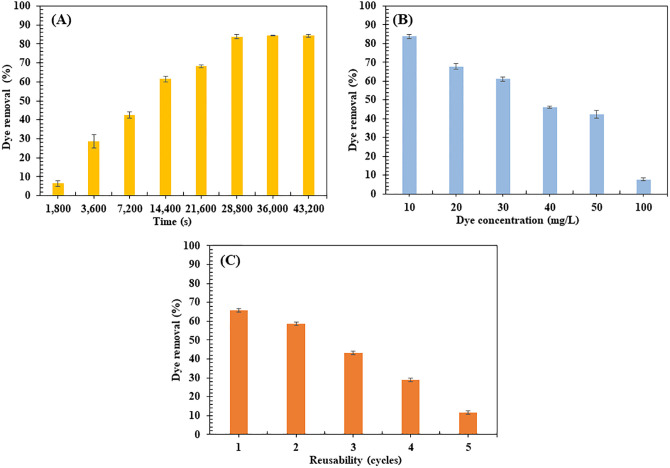
Congo Red (CR) dye removal percentage in wastewater using the biocomposite membrane (BM) shaken at room temperature (pH 6.7 and 150 rpm). (A) Effect of contact time (CR dye concentration used was 10 mg/L); (B) Maximum CR dye concentrations (10–100 mg/L) after 28,800 s of contact time and (C) Reusability: CR dye removal at a concentration of 10 mg/L over 28,800 s.

The evaluation of BM’s treatment performance for CR dye removal revealed several key findings about its adsorption capabilities and reusability (**Fig 4**). Initial experiments with contaminated water containing 10 mg/L CR dye showed a progressive increase in adsorption efficiency over time, reaching a maximum removal of 83.79 ± 1.09% after 28,800 s of treatment (**Fig 4A**). The rapid initial increase in dye removal can be attributed to the abundance of available active sites on the material’s surface. However, after 28,800 s, adsorption efficiency decreased as these sites became saturated with CR dye molecules, creating repulsive forces between adsorbed and solution-phase dye molecules [82,83]. This pattern of decreasing decolorization rate over time aligns with observations from previous studies [84,85].

When comparing BM’s performance with other composite or membrane materials, several trends emerge. Among the materials reviewed, ZIF-8@CS composites [86] exhibited the highest CR dye removal efficiency at 100%, followed by PVA/SA/ZSM-5-zeolite membranes [57] (99.9%) and CuSnO₂TiO₂ nanocomposites [87] (99%). Other materials, such as CS-VTM [88], or AM-CTS [72] composites, and clay-biomass composites [71], also demonstrated strong adsorption capabilities, with removal efficiencies ranging from 80% to 99%, depending on their composition. Although BM’s maximum removal efficiency of 83.8% is slightly lower than that of some advanced composites and membranes, it remains highly competitive due to its sustainable and cost-effective nature. Unlike synthetic materials that require complex fabrication, BM, derived from natural biomass such as cellulose from SCB and PHBV, presents an eco-friendly and biodegradable alternative for dye removal applications. While composite materials have been continuously developed, most are designed for the removal of dyes that are easier to degrade. As a result, the use of advanced materials for CR removal remains limited. This study fills that gap, expanding the options for dye wastewater treatment by offering an efficient, cost-effective, and environmentally friendly alternative with promising future applications.

Further investigation of the BM’s performance under varying dye concentrations (10–100 mg/L) at 28,800 s revealed a significant impact on removal efficiency. As dye concentration increased from 10–100 mg/L, the decolorization rate decreased substantially from 83.79 ± 1.09% to 7.69 ± 0.77% (**Fig 4B**). This decline resulted from the saturation of available adsorption sites and increased competition among dye molecules [14]. For comparison, previous research using activated carbon-incorporated tragacanth gum hydrogel biocomposites showed 96% dye removal at 25–100 mg/L, though efficiency dropped to 50% at 800 mg/L [89].

The reusability of BM was assessed through multiple adsorption cycles, with the material maintaining approximately 58.75 ± 2.79% and 43.31 ± 3.03% of its initial adsorption capacity after two and three cycles, respectively (**Fig 4C**). While this gradual decrease in performance suggests some degradation of adsorption capacity due to site blocking or depletion [90,91], the BM’s ability to maintain significant adsorption capability over multiple cycles demonstrates its potential for sustainable wastewater treatment applications. This reusability aspect is particularly important for practical applications, as it directly impacts the material’s long-term cost-effectiveness and sustainability [3,47].

#### Vegetable oil removal efficiency.

**Fig 5:**
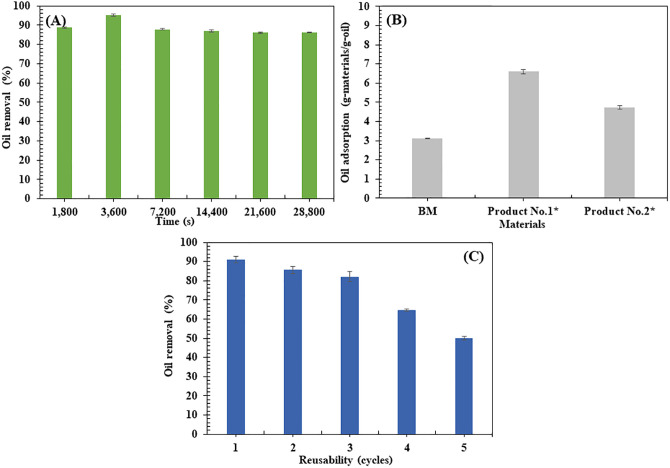
Vegetable oil (VO) removal percentage in water using biocomposite membrane (BM) shaken at room temperature (150 rpm). (A) Effect of time (VO concentration used was 4,000 mg/L); (B) Comparison of BM with common absorbent materials (*) and (C) Reusability: VO adsorption at a concentration of 4,000 g/L (3,600 s).

The BM demonstrated distinct patterns in oil removal efficiency and reusability. Using 4,000 mg/L VO, rapid adsorption occurred within the first 1,800 s, achieving 88.75 ± 0.35% removal. The efficiency peaked at 95.15 ± 0.49% after 3,600 s before stabilizing at 86.30 ± 0.28% at 28,800 s (Fig 5A).

This pattern reflects initial rapid uptake due to abundant vacant surface sites, followed by decreased adsorption as sites become saturated. The slowing rate can be attributed to repulsive forces from adsorbed oil molecules and the increased difficulty of remaining oil molecules to access deeper intraparticle sites [65,92–94].

Comparative analysis with commercial absorbents showed that BM’s oil absorption capacity (3.11 ± 0.03 g-oil/g-sorbent) was lower than both Product No. 1 (6.60 ± 0.12 g-oil/g-sorbent) and Product No. 2 (4.73 ± 0.10 g-oil/g-sorbent) (**Fig 5B**). However, BM exhibited superior reusability, maintaining 50.05 ± 1.06% of its initial absorbent capacity after five usage cycles (**Fig 5C**). This sustained performance across multiple cycles demonstrates our BM’s potential for practical applications, with its reusability offering advantages through reduced material waste and replacement needs. In comparison to previous studies, the developed BM showed a lower initial oil absorption capacity (3.11 g/g) than SCB polyurethane composites (15.2 g/g) [41] and hydrophobically functionalized biocomposites from lignocellulosic biomass [77], both of which demonstrated higher oil sorption efficiencies. Furthermore, the study on cellulose modified with amino-silane demonstrated the highest oil absorption after 604,800 s of testing [95]. While this demonstrates significant improvement in hydrophobicity through surface modification, the extended absorption time of 604,800 s can be a significant drawback compared to the much faster absorption of our BM, which only takes 3,600 s.

Although the BM’s maximum oil absorption capacity is lower than that of some commercial adsorbents, its excellent reusability, sustainability, and rapid adsorption kinetics make it a highly promising candidate for long-term, cost-effective, and environmentally friendly applications. The quick regeneration ability also makes it particularly suitable for time-sensitive oil spill remediation scenarios, where rapid performance is essential. These findings are consistent with previous reports on bio-based oil adsorbents [96–98], which emphasize the importance of reusability, rapid adsorption, and sustainable material design in effective oil spill management.

### Characterization of biocomposite membrane by FTIR and SEM

#### Surface analysis after Congo red dye adsorption.

**Fig 6:**
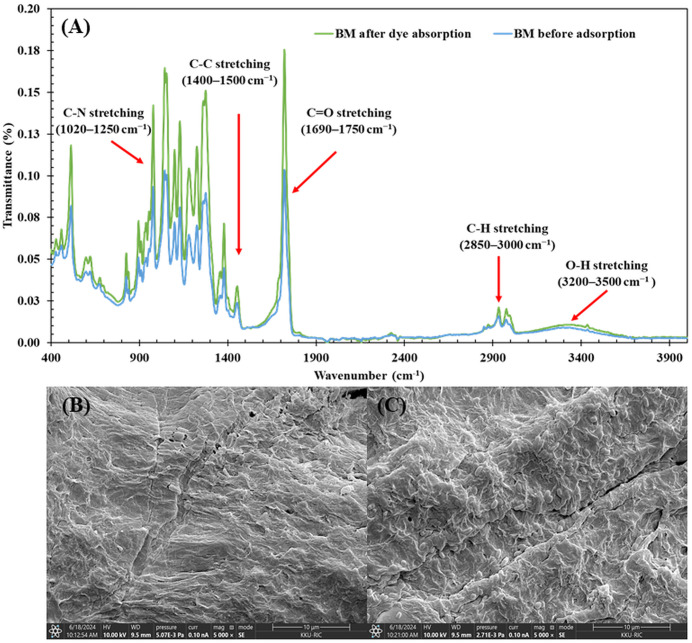
Characterization of the biocomposite membrane (BM) before and after adsorption of Congo Red (CR) dye under optimal adsorption conditions (CR dye concentration of 10 mg/L with 28,800 s). (A) FTIR spectra showing the functional groups of the BM before and after CR dye adsorption; (B) SEM image of the BM surface before CR dye adsorption and (C) SEM image of the BM surface after CR dye adsorption at a magnification of 5,000x.

FTIR analysis of BM after dye absorption revealed notable changes in functional group intensities, reflecting interactions during the adsorption process (**Fig 6A**). The most prominent changes were observed in C-H stretching (2850–3000 cm ⁻ ¹), which increased substantially by 711.08%, and aromatic C-C stretching (1400–1500 cm ⁻ ¹), which increased by 206.07%, suggesting that hydrophobic interactions and π-π interactions play dominant roles in dye adsorption. The C = O stretching (1690–1750 cm ⁻ ¹) showed a moderate increase of 53.23%, indicating active involvement of carbonyl groups with the dye. The O-H group (3200–3500 cm ⁻ ¹) decreased by 26.85%, suggesting some reduction in available hydrogen bonding sites.

In contrast, minimal changes were observed in N-O asymmetric stretching (1470–1550 cm ⁻ ¹, −13.46%) and aliphatic C-N stretching (1020–1250 cm ⁻ ¹, + 151.40%). Overall, these spectral changes suggest that dye adsorption on BM involves primarily hydrophobic interactions, π-π interactions, and hydrogen bonding, with these functional groups playing key roles in the adsorption process [99,100].

The analysis of surface morphology through SEM revealed clear changes in the BM structure before and after dye adsorption. Before adsorption, the BM surface exhibited numerous pores and a rough texture, with visible fibers (**Fig 6B**). These features enhance dye adsorption by increasing the surface area and providing more active sites for the reactive dye molecules to interact with. After dye adsorption, the surface became noticeably smoother (**Fig 6C**), and dark spots appeared, indicating successful incorporation of dye molecules into the pores of the adsorbent. These changes suggest that the dye molecules were effectively captured, leading to pore blockage and a reduction in surface roughness. Similar morphological modifications have been observed in other studies, where dye adsorption led to pore filling and surface smoothing, thereby reducing the material’s overall porosity [3,84,101]. This surface transformation reflects the interaction between the dye molecules and the functional groups on the BM, consistent with the adsorption process driven by both physical and chemical forces, as seen in similar studies [100,102].

#### Surface analysis after vegetable oil adsorption.

**Fig 7:**
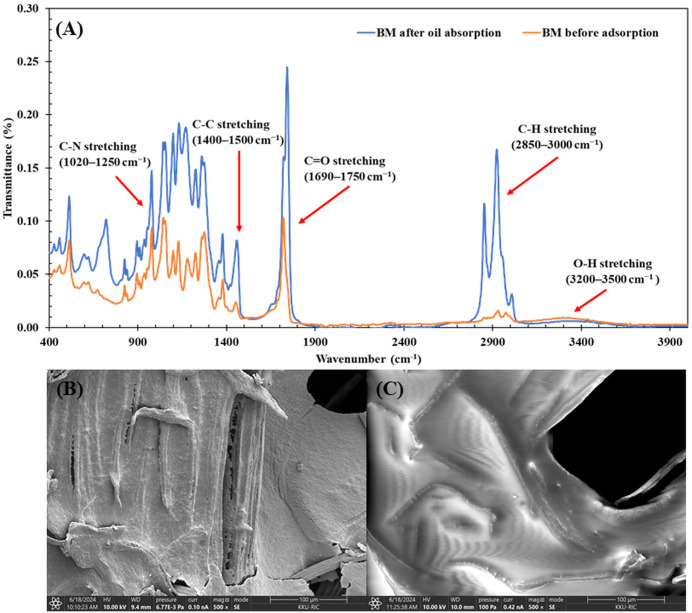
Characterization of the biocomposite membrane (BM) before and after vegetable oil (VO) adsorption under optimal conditions (VO concentration of 4,000 mg/L, 3,600 s contact time). (A) FTIR spectra, showing the functional groups of the BM before and after VO adsorption; (B) SEM image of the BM surface before VO adsorption and (C) SEM image of the BM surface after VO adsorption at a magnification of 5,000x.

FTIR spectra before and after VO adsorption (**Fig 7A**) revealed consistent peak locations with varying intensities after adsorption. The most substantial change was observed in C = O stretching intensity (+69.21%), highlighting strong interactions between carbonyl groups in the BM and VO, likely through dipole-dipole or hydrogen bonding. The aliphatic C-N stretching (1020–1250 cm ⁻ ¹) increased notably by 60.01%, and aromatic C-C stretching (1400–1500 cm ⁻ ¹) increased by 39.44%, indicating participation of amine groups and aromatic structures in adsorption. The O-H stretching (3200–3500 cm ⁻ ¹) increased moderately by 26.59%, suggesting enhanced hydrogen bonding between the BM and VO. In contrast, minor changes were observed in alkene C-H stretching (2850–3000 cm ⁻ ¹, + 6.48%) and N-O asymmetric stretching (1470–1550 cm ⁻ ¹, −4.23%), suggesting limited involvement of these groups in the adsorption process.

Overall, the FTIR results indicate that hydrogen bonding and van der Waals interactions are the primary mechanisms driving VO adsorption on the BM, supporting the physical adsorption model described by Al-Najar et al [56].

SEM analysis revealed significant changes in the BM surface morphology after VO adsorption. Before adsorption, the surface (**Fig 7B**) appeared rough and porous, providing a large surface area for oil absorption. After 3,600 s of contact time, the surface texture was completely transformed (**Fig 7C**), with distinct oil patches clearly visible, indicating successful oil adsorption. These oil patches suggest that the adsorbed oil molecules were effectively captured within the surface pores. This aligns with the hypothesis that PHBV contributes to the adsorption process due to its hydrophobic properties [103], which facilitate interactions between the oil and the biomass surface, enhancing oil sorption. These observations are consistent with previous studies, where hydrophobic interactions were identified as the primary mechanism for oil capture in similar biomaterials [31,104,105]. The SEM images further confirm that the surface changes, including oil patch formation, are indicative of successful oil adsorption driven by the material’s hydrophobic nature.

### Kinetic and isotherm study of biocomposite membrane on adsorption

#### Dye adsorption.

**Fig 8:**
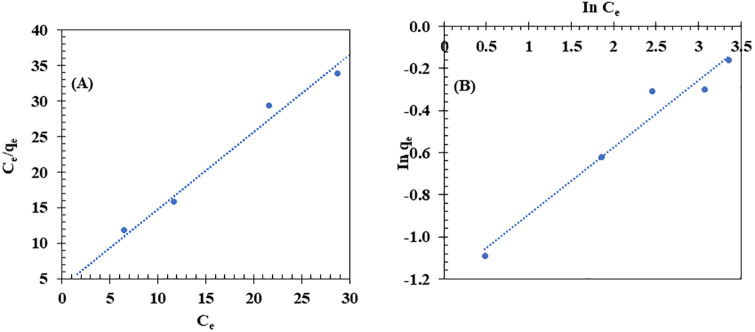
Langmuir (A) and Freundlich (B) isotherm plots for the adsorption of Congo Red (CR) dye onto the biocomposite membrane (BM). The experimental conditions included initial CR dye concentrations ranging from 10 to 50 mg/L, a contact time of 28,800 s, pH = 6.7, room temperature, and shaking at 150 rpm.

The applicability of the Langmuir and Freundlich models to describe CR dye adsorption onto BM was evaluated by fitting the experimental data to both models (**Fig 8**). As shown in **Fig 8A**, the experimental data fit well with the Langmuir model, exhibiting a high correlation (R² = 0.9869). The calculated equilibrium adsorption capacity (q_e_ = 0.92 mg/g) was obtained from the Langmuir model using the fitted parameters q_m_ and K_L_. This value is close to the experimental q_e_ (0.85 mg/g) at 10 mg/L CR, because the BM adsorption sites were nearly saturated under these conditions. Hence, q_e_, calc is approximately equal to q_m_, the maximum monolayer adsorption capacity. While the Freundlich isotherm (R² = 0.9632) (**Fig 8B**) also provided a good fit, the Langmuir model remains the preferred model for describing the adsorption behavior.

**Fig 9:**
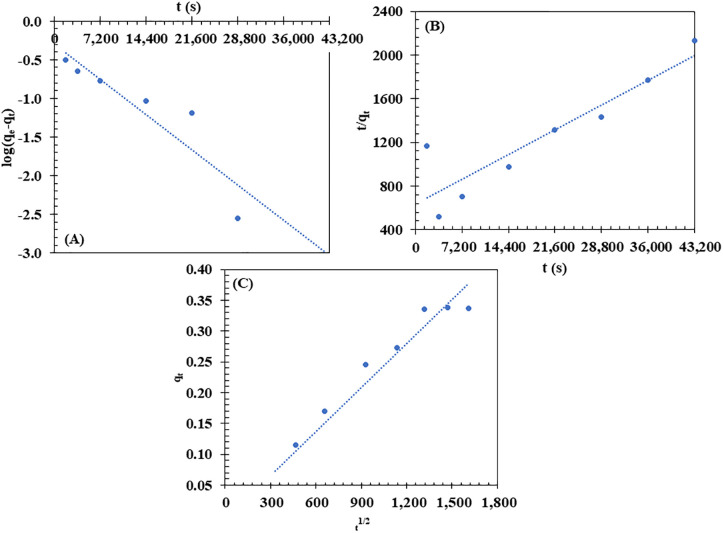
Plots of the pseudo-first-order (PFO), pseudo-second-order (PSO), and intra-particle diffusion models for Congo Red (CR) dye adsorption onto the biocomposite membrane (BM). Experimental conditions: initial CR concentration of 10 mg/L, contact time of 1,800–43,200 s, pH = 6.7, room temperature, with shaking at 150 rpm.

The kinetics of CR dye adsorption onto the BM were assessed by fitting the experimental data to the linearized forms of the PFO, PSO, and IPD models (**Fig 9**). The data showed a strong fit with the IPD model (R² = 0.9363) (**Fig 9C**), with an intra-particle diffusion rate constant (Kid) of 0.0143 mg g ⁻ ¹ s ⁻ ¹/². The high R² value suggests that the adsorption process is primarily controlled by diffusion within the pores of the biocomposite, likely due to its unique characteristics, such as its porous structure and the combination of cellulose and PHBV. These properties facilitate the diffusion process, making intra-particle diffusion the main mechanism for CR dye adsorption onto the BM.

In comparison, the PFO model exhibited a lower R² value of 0.8319 (**Fig 9A**), and the PSO model had an R² value of 0.8280 (**Fig 9B**). Although these models provided some degree of fit, their lower R² values indicate that they are less accurate in describing the adsorption kinetics. Therefore, the results suggest that intra-particle diffusion is the dominant mechanism in the adsorption of CR dye onto the BM.

#### Oil adsorption.

**Fig 10:**
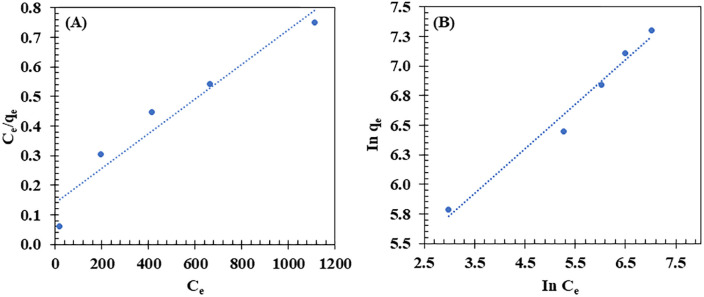
Langmuir (A) and Freundlich (B) isotherm plots for the adsorption of vegetable oil (VO) onto the biocomposite membrane (BM). Experimental conditions: initial VO concentration of 2,000 to 10,000 mg/L, contact time of 3,600 s, room temperature, and shaking at 150 rpm.

The applicability of the Langmuir and Freundlich models to describe VO adsorption onto the BM was evaluated by fitting the experimental data into both models (**Fig 10**). As shown in the results, the experimental data fit well with the Freundlich model (**Fig 10A**), exhibiting a high correlation (R² = 0.9784). The value of the Freundlich constant (n) was found to be 2.66, indicating a favorable adsorption process. This suggests that the Freundlich model is particularly suitable for describing oil adsorption onto the BM, as it reflects the multilayer adsorption process characteristic of the Freundlich isotherm. In contrast, the Langmuir model showed a slightly lower correlation (R² = 0.9367) (**Fig 10B**), indicating that while it can still describe the oil adsorption process, it is less applicable in this case. The Langmuir model typically describes monolayer adsorption, which may not fully capture the complexity of the adsorption in this study. Similar results were reported by Rengga et al. [106], where the adsorption of waste cooking oil by activated carbon from banana peel biomass was found to follow the Freundlich model with a high correlation (R² = 0.9700), reflecting a physical adsorption process.

**Fig 11:**
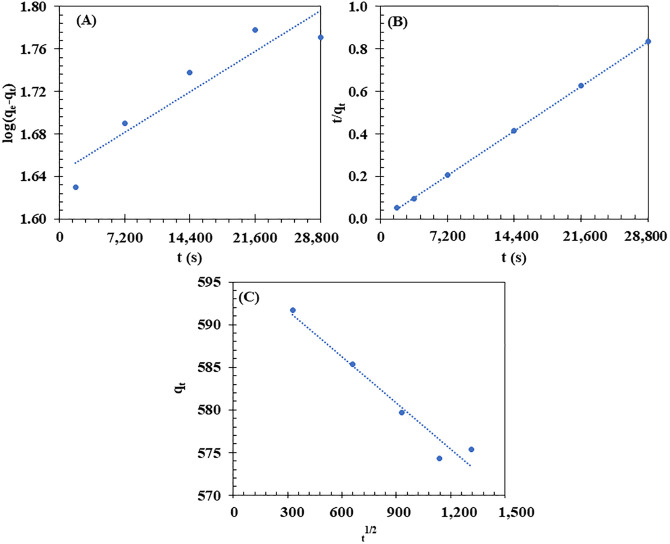
Plots of the pseudo-first-order (PFO), pseudo-second-order (PSO), and intra-particle diffusion models for vegetable oil (VO) adsorption onto the biocomposite membrane (BM). Experimental conditions: initial VO concentration of 4,000 mg/L, contact time 1,800–28,800 s, room temperature, shaking at 150 rpm.

The adsorption kinetics of VO onto the BM were analyzed using the PFO, PSO, and IPD models (**Fig 11**). The PSO model demonstrated the highest correlation (R² = 0.9999) (**Fig 11B**), indicating that chemisorption governs the process through valency forces or electron exchange. However, the calculated adsorption capacity (q_e_, cal. 555.56 mg/g) was much higher than the experimental value (q_e_, exp. 0.338 mg/g), suggesting that while the PSO model fits statistically, factors such as limited active sites or mass transfer constraints may reduce the actual adsorption efficiency. The IPD model also showed a strong correlation (R² = 0.9552) (**Fig 11C**), indicating that diffusion within the porous biocomposite structure, composed of cellulose and PHBV, significantly contributes to the adsorption process. The hydrophilic and hydrophobic domains of the material likely enhance VO retention. In contrast, the PFO model exhibited a lower correlation (R² = 0.8710) (**Fig 11A**), suggesting that physical interactions play a minor role. These findings highlight that VO adsorption onto the BM is controlled by both chemisorption and IPD. The PSO model showed the highest correlation, consistent with findings from Abel et al. [107] and Askari et al. [108], where the PSO model was also found to be the best fit for oil adsorption.

Overall, VO adsorption on the BM is driven by both physical (hydrogen bonding and van der Waals) and chemical (chemisorption and intraparticle diffusion) interactions, reconciling the apparently different conclusions from FTIR and kinetic analyses. This is consistent with previous studies showing that adsorption often involves simultaneous physical and chemical mechanisms, with intraparticle diffusion contributing to mass transfer alongside chemisorption [109].

Although direct thermodynamic parameters (ΔG°, ΔH°, ΔS°) were not calculated due to single-temperature experiments, the observed adsorption behavior of both CR dye and VO onto the BM can be qualitatively interpreted based on literature findings for analogous cellulose-based systems. For CR, similar cellulose-based adsorbents have shown spontaneous (negative ΔG°) and endothermic (positive ΔH°) adsorption, dominated by physisorption mechanisms such as hydrogen bonding, π–π interactions, and van der Waals forces [110–112]. For VO, comparable systems have exhibited spontaneous (negative ΔG°) and exothermic (negative ΔH°) adsorption, driven by both physical and chemical interactions [113]. These literature observations suggest that adsorption of both CR and VO onto the BM may be energetically favorable, supporting the proposed adsorption mechanisms, though direct thermodynamic measurements would be needed for definitive characterization of our specific system.

### Adsorption mechanisms of dye and oil on biocomposite membrane

**Fig 12:**
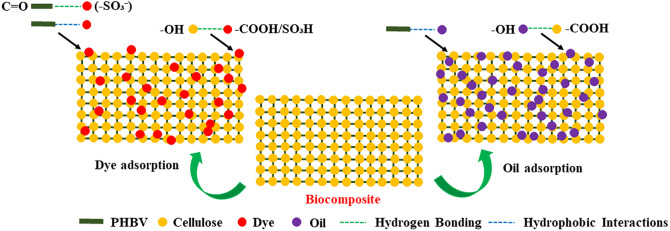
Proposed mechanism of adsorption of Congo Red (CR) dye and vegetable oil (VO) onto the biocomposite membrane (BM).

The adsorption mechanisms of CR dye and VO by the BM, synthesized from cellulose and PHBV, involve complex chemical interactions that work synergistically to enhance overall adsorption efficiency (**Fig 12**). The cellulose component, rich in hydroxyl groups (-OH), facilitates the adsorption of polar and moderately non-polar molecules through distinct mechanisms. Primary among these is hydrogen bonding, where cellulose’s -OH groups form bonds with polar functional groups on CR dye molecules, including -NH_2_, -SO_3_H, and -COOH, significantly enhancing the binding and immobilization of CR molecules [75,76]. Similarly, cellulose interacts with VO through hydrogen bonding with polar components like free fatty acids and glycerol, while also engaging with non-polar components such as triglycerides and hydrocarbon chains through hydrophobic interactions and van der Waals forces [104,114].

PHBV, the hydrophobic biopolymer component, plays a crucial complementary role in capturing non-polar substances. Its molecular structure, characterized by long polyester chains lacking polar functional groups, enables effective hydrophobic interactions with non-polar molecules in VO and hydrophobic components of CR dye molecules. These interactions, driven by van der Waals forces, significantly enhance BM’s capacity to adsorb water-insoluble molecules [3,115]. The integration of cellulose and PHBV in the BM creates a powerful synergistic system where each component enhances the other’s adsorption capabilities. While cellulose predominantly binds polar substances, such as functional groups in CR dye and polar components in VO, PHBV targets non-polar molecules, including hydrocarbons in VO and hydrophobic regions of CR dye. This complementary interaction results in a versatile and highly efficient adsorption capacity, making the BM particularly effective for diverse water treatment applications.

### Influence of ionic salts and synthetic wastewater on dye and oil adsorption by biocomposite membrane

#### Ionic salts on adsorption.

**Fig 13:**
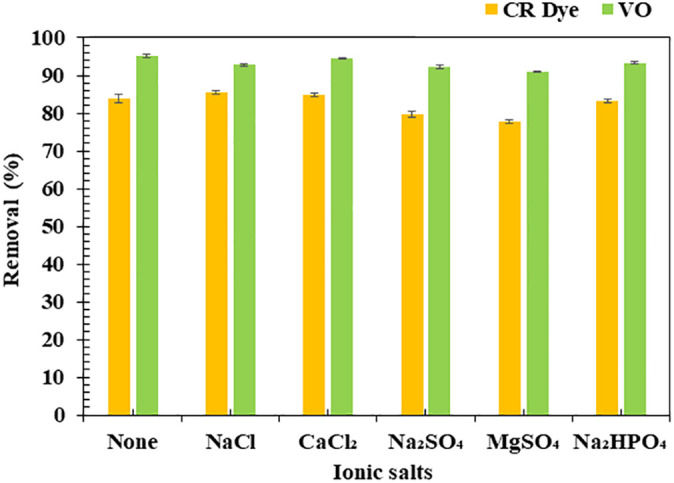
Adsorption efficiency of Congo Red (CR) dye and vegetable oil (VO) onto the biocomposite membrane (BM) in the presence of different ionic salts. Experimental conditions: CR dye adsorption: 10 mg/L CR dye, 28,800 s contact time, pH 6.7. VO adsorption: 4,000 mg/L VO, 3,600 s contact time. All experiments were conducted at room temperature with shaking at 150 rpm.

The adsorption efficiency of CR dye and VO onto the BM was evaluated in the presence of various ionic salts, including NaCl, CaCl₂, Na₂SO₄, MgSO₄, and Na₂HPO₄ (**Fig 13**). Monovalent ions (Group I, e.g., Na⁺) and divalent ions (Group II, e.g., Ca² ⁺ , Mg²⁺) reduced CR dye removal due to ionic interactions and competition for adsorption sites, with MgSO₄ having the least effect and NaCl/CaCl₂ showing similar influence. In contrast, VO adsorption remained consistently high (>90%) under all conditions, indicating the BM’s robustness for oil adsorption even in saline environments. These results align with previous studies demonstrating that salts can diminish dye adsorption but have minimal effect on nonpolar oil adsorption [60,116].

#### Biocomposite membrane adsorption efficiency under synthetic wastewater conditions.

**Fig 14:**
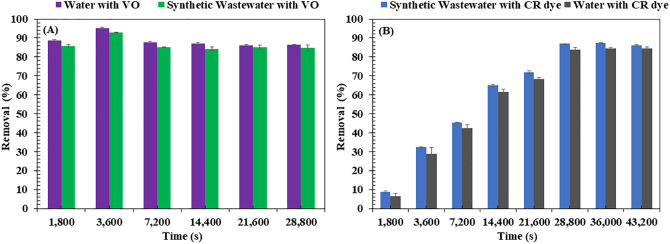
Adsorption efficiency of Congo Red (CR) dye and vegetable oil (VO) onto the biocomposite membrane (BM) in synthetic wastewater. Experimental conditions: CR dye adsorption initial concentration of 10 mg/L; VO adsorption initial concentration of 4,000 mg/L. All experiments were conducted at room temperature with shaking at 150 rpm.

The adsorption efficiency of the BM for CR dye and VO was evaluated under highly saline and alkaline synthetic wastewater conditions (pH 9.7), providing key insights into its performance (**Fig 14**). For VO removal, the BM maintained a high adsorption efficiency of 95.15 ± 0.5% within 3,600 s in oil-contaminated water (**Fig 14A**). In synthetic wastewater with elevated pH, the efficiency slightly decreased to 92.96 ± 0.2%, likely due to modifications in the oil’s chemical properties or changes in the BM’s surface characteristics under alkaline conditions [116]. Conversely, the high pH condition enhanced CR dye adsorption. In synthetic wastewater, its adsorption efficiency increased from 83.79 ± 1.0% to 86.90 ± 0.2% over 28,800 s (**Fig 14B**). This improvement is likely due to structural changes in the CR dye at high pH, which may have strengthened electrostatic interactions [117] with the BM, thereby increasing adsorption capacity. These findings underscore the BM’s strong potential for wastewater treatment applications, particularly in alkaline and saline environments such as textile effluents or marine areas impacted by petrochemical spills. Its stable performance across varying conditions highlights its effectiveness in treating both organic pollutants and dyes in real-world wastewater scenarios.

### Comparative analysis of biocomposite performance

The BM exhibited a maximum adsorption capacity (Qmax) of 0.92 mg/g for CR dye under the experimental conditions (10 mg/L, pH 6.7, 150 rpm, room temperature). While this Qmax is lower than those reported for some other composites, it remains competitive for dye adsorption applications. For instance (**Table 1**), the PVA/SA/ZSM-5 zeolite composite showed a Qmax of 5.33 mg/g [57], likely due to the high surface area of ZSM-5 providing extensive adsorption sites. Similarly, the ZIF8@CS and CuSnO₂TiO₂ composites exhibited Qmax values of 922 mg/g [86] and 277 mg/g [87], respectively, highlighting the strong adsorption potential of metal oxide- and zeolite-based materials. The relatively lower Qmax of the BM may be attributed to two main factors: (1) the use of a low initial dye concentration (10 mg/L) in this study compared to 50–100 mg/L or higher in other studies, which inherently limits the amount of dye that can be adsorbed at equilibrium, and (2) the material properties, which offer fewer adsorption sites compared to advanced zeolite- or metal oxide-based composites. However, its biodegradability, ease of preparation, and environmental safety make it an attractive option for sustainable applications. Notably, the clay-biomass composite exhibited a Qmax over 119 mg/g [71], further emphasizing the potential of biomass-based materials for dye removal. Future studies should explore higher initial dye concentrations and potential surface modifications to fully characterize and enhance the adsorption capacity of the BM.

**Table 1:** Comparison of CR dye adsorption efficiency among various composite materials.

Composite	Conditions	Qmax, mg/g
**PVA/SA/ZSM-5 zeolite [57]**	CR dye 10 mg/L, pH 3, stirred at room temperature	5.33
**CuSnO_2_TiO_2_ [87]**	pH 7, dye 20 ppm, 7,200 s	277
**Clay-Biomass [71]**	Azo dyes 50 mg/L, pH 2, stirred (160 rpm) at room temperature (3,600 s)	119.7
**ZIF8@CS [86]**	CR dye 100 mg/L within 10,800 s	922
**AM-CTS [72]**	CR dye 50 mg/L, pH 3, stirred (200) at 55°C (2,700 s)	104
**CS-VTM [88]**	CR dye 100 mg/L, pH 6 (65°C)	62.2
**AF-S [118]**	CR dye 100 mg/L, pH 5 (14,400 s)	28.24
**BM (This study)**	CR dye 10 mg/L, pH 6.7, stirred (150 rpm) at room temperature (28,800 s)	0.92

Another key advantage of the BM, beyond its safety, sustainability, and cost-effectiveness, is its ability to adsorb both dyes and oils. It exhibited an oil absorption capacity of 3.13 g-oil/g-sorbent, which, while lower than other composites such as hydrophobic biocomposite (18 g-oil/g-sorbent) and carbon-polyethylene terephthalate (16 g-oil/g-sorbent) (**Table 2**), remains a viable alternative. Notably, the BM retained over 50% of its efficiency after five cycles of oil absorption, demonstrating excellent regeneration capacity and long-term usability. This dual adsorption capability enhances its potential for environmental applications, including wastewater treatment and oil spill cleanup. The results highlight a trade-off between adsorption efficiency and environmental impact, reinforcing the value of BM as a versatile and sustainable solution for diverse environmental challenges. However, further research should focus on optimizing efficiency while maintaining sustainability, environmental friendliness, and cost-effectiveness.

**Table 2:** Comparison of oil adsorption efficiency among various composite materials.

Composite	Absorption capacity (g-oil/g-sorbent)
**Hydrophobic Biocomposite [4]**	18
**Saw Dust–Bentonite–Ca(OH)_2_ Mixture [119]**	16
**Sugarcane Bagasse-Polyurethane Composite [41]**	15.2
**Carbon-Polyethylene Terephthalate [120]**	8
**BM (This study)**	3.11

## Conclusion

This research successfully developed a BM from SCB-derived cellulose fiber and PHBV for water remediation, with CR dye and VO adsorption evaluated separately. The BM demonstrated excellent adsorption capabilities, achieving removal efficiencies of 83.79% for CR dye and 95.15% for VO, with significant reusability over multiple cycles. Isotherm studies revealed that CR adsorption followed the Langmuir model, while VO adsorption aligned with the Freundlich model. Kinetically, the IPD model best described CR adsorption, whereas the PSO model provided the best fit for VO adsorption. The BM exhibited a neutral surface charge (pH_PZC_ of 7.29 ± 0.04) and maintained stable performance in the presence of ionic salts and synthetic wastewater, demonstrating its practical applicability. Despite having lower oil absorption capacity than some commercial sorbents, the BM’s advantages in reusability, biodegradability, and cost-effectiveness position it as a promising sustainable alternative for wastewater treatment, aligning with circular economy principles. Future research should focus on: (1) conducting multi-temperature experiments to determine thermodynamic parameters (ΔG°, ΔH°, ΔS°) for deeper mechanistic understanding; (2) exploring higher dye concentrations (50–200 mg/L) to fully characterize maximum adsorption capacity; (3) testing performance with real industrial wastewaters at larger scales; (4) optimizing BM composition through surface modifications to enhance adsorption capacity; and (5) developing efficient regeneration methods and cost-effective production processes for industrial-scale applications.
